# An AGM Model for Changes in Complement during Pregnancy: Neutralization of Influenza Virus by Serum Is Diminished in Late Third Trimester

**DOI:** 10.1371/journal.pone.0112749

**Published:** 2014-11-19

**Authors:** Anne E. Mayer, Griffith D. Parks

**Affiliations:** Department of Microbiology and Immunology, Wake Forest School of Medicine, Winston-Salem, NC 27101, United States of America; University of California Riverside, United States of America

## Abstract

Pregnant women in the third trimester are at increased risk of severe influenza disease relative to the general population, though mechanisms behind this are not completely understood. The immune response to influenza infection employs both complement (C′) and antibody (Ab). The relative contributions of these components to the anti-viral response are difficult to dissect because most humans have pre-existing influenza-specific Abs. We developed the African green monkey (AGM) as a tractable nonhuman primate model to study changes in systemic innate immunity to influenza during pregnancy. Because the AGMs were influenza-naïve, we were able to examine the role of C′ in influenza virus neutralization using serum from non-pregnant animals before and after influenza infection. We determined that serum from naïve AGMs neutralized influenza via C′, while post-infection neutralization did not require C′, suggesting an Ab-mediated mechanism. The latter mimicked neutralization using human serum. Further, we found that *ex vivo* neutralization of influenza with both naïve and influenza-immune AGM serum occurred by virus particle aggregation and lysis, with immune serum lysing virus at a much higher rate than naïve serum. We hypothesized that the anti-influenza C′ response would diminish late in AGM pregnancy, corresponding with the time when pregnant women suffer increased influenza severity. We found that influenza neutralization capacity is significantly diminished in serum collected late in the third trimester. Strikingly, we found that circulating levels of C3, C3a, and C4 are diminished late in gestation relative to nonpregnant animals, and while neutralization capacity and serum C3a return to normal shortly after parturition, C3 and C4 levels do not. This AGM model system will enable further studies of the role of physiologic and hormonal changes in downregulating C′-mediated anti-viral immunity during pregnancy, and it will permit the identification of therapeutic targets to improve outcomes of influenza virus infection in pregnant women.

## Introduction

Pregnancy is a unique physiologic state that is essential for the survival of all mammalian species. It requires the maternal immune system to perform the seemingly contradictory roles of tolerance to accept the fetus (as an alloantigen) and protection of the mother and fetus from infection and inflammatory insults. Pregnancy has historically been characterized as a state of immune suppression [1]; however, this description oversimplifies the complex physiologic requirements of the progression from implantation and placentation, to rapid fetal growth and development, to preparation for delivery [2]. These distinct phases of pregnancy have different physiologic needs and are accompanied by highly regulated hormone alterations, shifting profiles of energy expenditure, and a changing immunological milieu [3], [4]. Given these unique alterations, it stands to reason that pregnant women experience a distinct range of immunological challenges [2], [5], [6]. Together, this raises the clinically relevant question of how the innate immune response to viruses is altered during pregnancy [7].

Influenza virus infection is associated with a disproportionately high burden of disease in pregnant women around the world, during both pandemics and seasonal outbreaks [3], [8], [9]. During the 2009 H1N1 pandemic, for example, pregnant women were at ten-times the risk of severe infection relative to non-pregnant women [9]. There has been extensive documentation of pregnant women infected with influenza virus suffering disproportionately severe respiratory disease and the public health impact of this phenomenon [10]–[12]. These severe influenza virus infections are most common late in pregnancy [9], [10], [13] and may lead to serious outcomes such as low gestational weight, preterm birth, and infant failure to thrive, even in the absence of fetal infection [9], [14], [15]. These effects pose significant public health challenges and are of great economic cost worldwide.

Though complement (C′) has been traditionally regarded as playing an important role in protection from bacterial infections, it also plays a significant role in the innate response to a number of viruses including VSV, mumps, measles, Newcastle disease, parainfluenza viruses, and influenza virus [16]–[21]. The C′ cascade has unique and essential roles in early responses to infection such as direct virus neutralization, induction of chemotaxis, enhancement of phagocytosis, immune complex clearance, and lysis of infected cells [22]. Specifically, C′ is essential for effective clearance of influenza virus [21], [23], [24]. For example, C3−/− mice are highly susceptible to influenza virus infection—with increased virus titers and delayed clearance relative to WT mice [23].

While natural antibodies (nAbs) play an important role in eliciting an effective immune response against primary infections with influenza virus [24], [25], natural IgM (nIgM) does not effectively neutralize influenza virus in the absence of C′ [21]. This also holds true for other types of low affinity Abs, such as cross-reactive Abs [26], [27]. Further, it has been shown that C′ can fix Ab to mediate opsonization or membrane attack, via the classical pathway. From a clinical context, seasonal influenza epidemics are comprised of disease among individuals previously infected with closely related strains in prior seasons; this indicates that cross-reactive Abs are often not adequate to protect healthy adults, even in the presence of normal C′. In fact, the role that cross-reactive Ab plays in protecting a patient from a new influenza virus infection is highly strain-dependent [28]. We hypothesize that differences in C′ between pregnant women and other healthy adult populations contribute to the increased disease severity seen in the pregnant population. Here we developed a model that enabled us to identify the unique and non-redundant roles of C′ in influenza virus neutralization with influenza-naïve serum.

While C′ effects indirectly enhance the downstream immune response (e.g. by increasing phagocytosis and acting as chemoattractants for innate immune cell types), we have more recently come to appreciate that the C′ system can also directly regulate humoral immunity and T cell functions [29]–[31]. Therefore, alterations in C′ levels and/or function have the potential for broader effects on the whole immune response to a pathogen. The role of C′ in increased severity of influenza during pregnancy, however, has not been previously explored.

C′ activation is correlated with poor pregnancy outcomes such as pre-eclampsia and preterm birth, leading to the proposal that C′ inhibition is an “absolute requirement” of normal pregnancy [32]. It has been proposed that C′ activation during pregnancy leads to a pro-inflammatory, pro-coagulant, and tissue damaging environment surrounding the fetus [33]. For example, high levels of anaphylatoxins, such as C5a, induce the release of potent anti-angiogenic factors, which sequester growth factors that are essential for normal placental development and healthy pregnancy [34]–[36]. However, these studies seem at odds with a number of reports that C′ is activated during pregnancy [37]–[39]. Such disparities may reflect the difficulties inherent in performing longitudinal studies in pregnant women. Further, to our knowledge, no analysis of functional C′ activity against pathogens has been performed using samples from pregnant women. Thus, the question of how C′ functions against pathogens such as influenza virus during pregnancy remains unresolved.

Human studies pose significant obstacles to addressing this experimental question because of differences in anti-influenza virus antibody levels among subjects, which confounds the analysis of the role of C′ in neutralization [40], [41]. Thus, we sought to identify an influenza-naïve pregnancy model system that closely mimics the pregnancy physiology of women. Mice are the most common animal model used in pregnancy studies because they are relatively inexpensive, are reared in an easily controlled environment, and their short gestation period enables rapid experiments and large sample sizes. However, murine reproductive physiology differs greatly from that of humans [42], [43]. The African green monkey (AGM) is a strikingly suitable model for studies of reproductive physiology, with many characteristics in common with humans (Table 1). For example, AGMs have cycle types, hormone levels, and pregnancy physiology very similar to those of humans. Additionally, while AGM fetal development occurs in a similar fashion as humans, the 5.5-month AGM gestation period is slightly compressed and more accessible than that of humans. For this study, the Wake Forest University Primate Center (WFUPC) Vervet Research Colony (VRC) served as a source for groups of pregnant and control female AGMs. Access to the VRC enabled us to perform longitudinal sampling during and after pregnancy.

**Table 1:** Reproductive comparisons across species.

	Mouse^a^	Human^b^	AGM^c^
**Gestation Length**	18–20 days	∼280 days	∼155 days
**Number of Offspring per Parturition**	6–8	1–2	1–2
**Hormone Sources During Pregnancy**	Corpus luteum is source of progesterone; systemic progesterone withdrawal prior to parturition	Corpus luteum initial source; placenta after eighth week	Placenta is source of progesterone
**Structure of Female Reproductive Tract**	Duplex (two separate uterine horns)	Simplex (single cavity) uterus	Simplex uterine cavity similar to human
**Cycling Estrogen Levels**	∼100 pg/ml (estrous peak)	∼400 pg/ml (preovulatory peak)	∼500 pg/ml (preovulatory peak)
**Cycle Type**	Estrous (4–5 d)	Menstrual (28 d)	Menstrual (31 d)
**Age at Onset of Puberty**	6 w	10–11 y	3 y

a See reference [76].

b See reference [77].

c See reference [78]–[84].

Here, we demonstrate the utility of this novel AGM model system by determining how C′ levels and the capacity for C′-mediated virus neutralization change during pregnancy. We found that late in third trimester (T3), pregnant animals have significantly diminished circulating C′ factors and decreased capacity to neutralize influenza virus. Our data support a model whereby these late T3 C′ reductions, which may be essential to permit the maintenance of healthy pregnancy, could increase the severity of influenza disease.

## Materials and Methods

### Cells, viruses, and serum

Monolayer cultures of MDBK cells [44] were grown in Dulbecco's modified Eagle medium (DMEM) supplemented with 10% heat-inactivated fetal bovine serum (FBS), penicillin (100 units/ml), streptomycin (100 ug/ml) and 2 mM L-Glutamine. U937 cells (ATCC, Manassas, VA) were grown in RPMI supplemented as above. Influenza A virus engineered to encode GFP as a fusion protein with the viral NS1 protein was the kind gift of Adolfo Garcia-Sastre [45] and was grown in 10 day old embryonated chicken eggs as described previously [46]. The properties of individual normal human sera (NHS) from healthy donors has been described previously [20]. Commercially available pooled human sera was from Complement Technology, Inc. (Tyler, TX).

### Ethics statements and animal husbandry

Individual NHS samples were collected following approval of the Wake Forest University IRB under human protocol number BG04–504 and written consent of the individuals. AGM studies were carried out in strict accordance with the recommendations in the Guide for the Care and Use of Laboratory Animals of the National Institutes of Health. The protocols were approved by the Wake Forest University Animal Care and Use Committee (A13–023; A13–027) and utilized animals from the Vervet Research Colony (VRC) of the Wake Forest University Primate Center (WFPC). At the time of the study, the colony consisted of 486 animals in their second to eighth generation in captivity. All animals were colony-born and were of known age. Regular procedures revolve around the tri-annual TB testing program, during which time every animal in the colony is captured, sedated, physically examined, and tested for TB, and (if female) given a reproductive ultrasound. In addition, an appropriate environmental enrichment SOP for the influenza infection project (A13–023) was approved by the Wake Forest University Nonhuman Primate Environmental Enrichment Committee. This included, but was not limited to: pair-housing the majority of the time, but single-housed temporarily for experimental requirements; monkeys located to have auditory, visual, and olfactory stimulation from other monkeys in the room; and monkeys received species-appropriate toys and manipulate, which were rotated regularly. All experimental procedures were performed only with sedated animals and appropriate analgesia was administered. Steps were taken to ameliorate suffering and improve animal welfare in accordance with the recommendations of the Weatherall report (2006). Pregnant, postpartum, and control animals for the pregnancy portion of this work were housed in large maternal social groups with indoor/outdoor housing and equipment for normal physical activity. All animals were fed the Purina #5038 normal protein monkey chow diet *ad libitum*. Food was removed the night before procedures requiring sedation and/or anesthesia. Animals in this project were fully under the care of veterinarians at the WFU School of Medicine in accordance with the standards incorporated in The Guide to the Care and Use of Laboratory Animals (2011).

### Animal infection

Healthy 18–22 yo African Green monkeys (*Chlorocebus aethiops*) were sedated with ketamine, and placed in a dorsal position before receiving 8×10^9^ 50% Egg Infective Dose (EID_50_) of purified H1N1 influenza strain A/Puerto Rico/8/1934 (PR8) delivered in 2 ml sterile PBS. One ml was delivered to the trachea and 0.5 ml was delivered to each nostril. Femoral blood was collected at the days pi indicated in the figure legends and allowed to clot for 45 min at 37°C. The clot was retracted and serum was separated by centrifugation, aliquoted and stored at −80°C. Complement inactivated serum was prepared by heating inactivation (HI) of normal AGM serum (NAGS) at 56°C for 30 min.

### Pregnant animals

Fifty-nine AGMs in the VRC were identified as pregnant between January and May 2012 and used in this study. Control animals were selected at random from the VRC during two dates overlapping with collection dates for pregnant animals. The animals used in the study are described in Table 2. We used third trimester and postpartum samples from a longitudinal sampling scheme that involved tracking and sampling of each animal two to three times during pregnancy, once within 5–10 days following parturition at check-in (PC), and once two to six months postpartum (PP). Repeated blood samples from the pregnant animals in the breeding colony were obtained during TB testing dates, following sedation and ultrasound confirmation of pregnancy status. Briefly, during the 2012 breeding season, femoral vein blood samples were collected from each pregnant animal during the regularly scheduled TB testing in January, May, and September. We faced the challenge of having no *a priori* knowledge of conception dates for females in the VRC colony. Ultrasound was used to estimate the gestational age of the fetus based on fetal head circumference [47]. Additional dates were scheduled in April and July 2012 for blood draws, physical exams, and ultrasounds to confirm healthy gestation. Serum was isolated and stored as described above. Samples used in C′ ELISAs and neutralization experiments were categorized based on the gestation date when the sample was collected.

**Table 2:** Characteristics of AGM pregnant and control samples.

	Pregnant	Controls
**Total number of animals**	53 (+6 prior to sampling)	24
**Age range**	3.3–17 years	5–20 years
**% primiparous**	18.9%	N/A
**% with history of poor outcomes** a	22.6%	N/A
**% with 2012 poor outcome** b	16.9%	N/A
**T1** c	13 (11.8%)^d^	N/A
**T2** c	37 (33.6%)^d^	N/A
**T3** c	60^e^ (54.5%)^d^	N/A
**Parturition check-in** c	58	N/A
**2–6 mo post partum** c	54	N/A

a Percent of animals that have had stillbirths, aborted fetuses, or infant mortality less than 6 months old in pregnancies prior to 2012.

b Same as a, but during 2012 breeding season when samples were collected.

c Number of animals (percent of animals in parentheses) for which we collected a sample during Trimester 1 (<55 days), Trimester 2 (55–110 days), Trimester 3 (>110 days), at parturition check-in (5–10 days after parturtion), and 2–6 months postpartum.

d Number in parentheses indicates percent of total pregnancy samples collected during indicated trimester.

e 7 animals (60–53 = 7) were sampled twice during T3.

### Serum neutralization assays

PR8 neutralization experiments were performed by treating 5 PFU of PR8-GFP with normal or HI human or AGM sera diluted in RPMI (in a total volume of 100 µl), as indicated in the figure legends for 45 min at 37°C. U937 cells (2×10^5^) were added, and the mixtures were analyzed 48 h later for GFP expression. This extended incubation period allowed for high sensitivity and detection of small differences in neutralization capacity across serum samples. The effective dose 50 (ED_50_) was developed as a measure representative of neutralization capacity for serum samples collected from pregnant and postpartum animals. Neutralization assays were carried out using different dilutions of sera, as described above. For each dilution (1∶40, 1∶80, 1∶160, and 1∶320) of each serum sample, the percent neutralization was calculated using the following equation:

% neutralization_(NAGS)_  =  [1- (%GFP^+^_(NAGS)_)/(%GFP^+^_(No serum)_)]*100

This number was plotted on the y-axis against the corresponding serum dilution on the x-axis, and a logistic regression was performed over the whole series, as shown in Results. In this fashion, a logistic regression was performed for samples from each animal, and the ED_50_ was determined by calculating the amount of serum required to achieve a y-value of 50% neutralization.

### Electron microscopy

Electron microscopy (EM) analysis of sucrose gradient-purified PR8 was carried out as previously described [20]. Briefly, 5 µl of a 1∶5 dilution of PR8 (13.8 µg of PR8) was incubated with 5 µl of a 1∶5 dilution of AGM serum at 37°C for the time period indicated in the figure legend. Zero min incubations were performed on ice. After the indicated time, mixtures were placed on Formvar carbon-coated 200-mesh gold grids (EM Sciences, PA) and incubated in a humidified chamber for 10 min before fixing with 2.5% glutaraldehyde. Samples were negatively stained with 2% phospho-tungstic acid and analyzed with a Technai transmission electron microscope.

### Western blotting

Western blotting was performed as described previously [48]. Specifically, either 1∶50, 1∶250, and 1∶500 dilutions of each NHS and NAGS samples (for species comparison) or 1∶200 and 1∶400 dilutions of each pregnancy NAGS sample (for quantification) were electrophoresed and transferred to a nitrocellulose blot which was probed with goat anti-human C3 primary Ab at a 1∶2000 dilution (Calbiochem), followed by HRP-conjugated donkey anti-goat at a dilution of 1∶4000. Quantities of AGM C3 were determined relative to 50 ng, 100 ng, and 200 ng samples of purified human C3 (Complement Technology, Inc., Tyler, TX) using a Kodak Image Station 4000R and Carestream MI software (Molecular Imaging System Carestream, Health Inc.).

### ELISA

MaxiSorp 96-well ELISA plates (Nalge Nunc International, Penfield, NY) were coated with 100 µl of diluted serum (1∶32,000 for C3; 1∶16,000 for C3a; 1∶6000 for C4), or with dilutions of purified C3, C3a or C4 human standards (all from Complement Technology). Plates were incubated at 4°C overnight, washed three times with PBS/Tween (0.2%), and wells were blocked with 200 µl of PBS containing 5% milk for 1 hr at 37°C. 100 µl of diluted primary goat anti-human C3 (1∶2000), rabbit anti-human C3a (1∶5000) or goat anti-human C4 (1∶2000) (all from Calbiochem) was added to each well and incubated for 1 h at room temperature (RT). Wells were washed five times with PBS/Tween before incubation at RT for 1 h with 100 µl per well of HRP-conjugated donkey anti-goat (1∶20,000 for C3 and C4) or goat anti-rabbit IgG (1∶50,000 for C3a) (Jackson ImmunoResearch Laboratories, PA) followed by development with substrate TMB (tetramethylbenzidine dihydrochloride, Sigma). The absorbance was determined at 450 nm on a ELx800 plate Absorbance Microplate Reader (Bio Tek Instruments, Inc., Vermont).

### Statistics

Graphpad Prism and was used to perform all analyses. The Shapiro-Wilk normality test was performed on each group. Based on these findings, two-tailed unpaired t-tests were performed on log-transformed neutralization percentages for comparisons between NAGS and NHS and between naïve and immune sample dates, as well as to compare C3 β-chain concentrations between control and T3 groups. In the comparison of Control vs T3 % serum for PR8 ED_50_, the T3 distribution did not pass the Shapiro-Wilk normality test, so the Mann-Whitney compare ranks two-tailed non-parametric test was used. A one-way ANOVA and Tukey correction for multiple comparisons was used for comparisons of greater than two categories. Mean and SD are shown as measures of dispersion for each group tested in every figure. p-values represented as: * p<0.05; ** p<0.001; *** p<0.0001.

## Results

### Comparison of C′ concentrations in human and AGM sera

**Figure 1:**
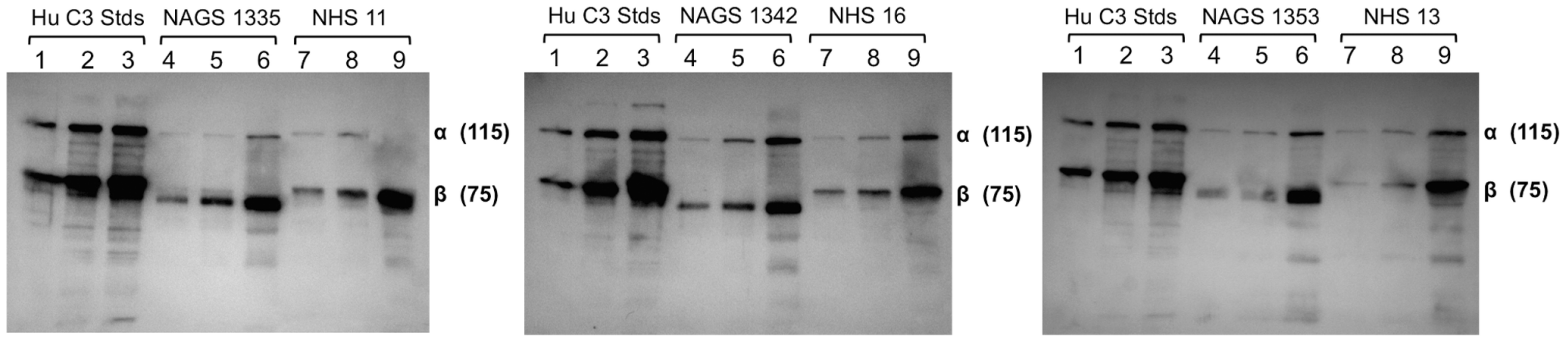
Comparison of C3 concentrations in NHS and NAGS. NAGS and NHS were used to compare C3 concentrations by western blot, relative to purified Hu C3 controls. Controls of 50 ng, 100 ng, and 200 ng are in lanes 1, 2, and 3, respectively, on each of the three blots. 1∶500, 1∶250, and 1∶50 dilutions of NAGS are in lanes 4, 5, and 6, respectively and of NHS are in lanes 7, 8, and 9, respectively. One NAGS donor and one NHS donor were on each blot, as indicated in the figure by NAGS or NHS number. C3 β- and C3 α-chain and their MW in kilodaltons are indicated on each blot.

To our knowledge, direct quantitative comparisons of serum C3 concentrations in humans and AGMs have not been previously published. To verify relevance of the AGM model for human C′ studies, three dilutions of NAGS (Fig. 1, lanes 4–6) or NHS (Fig. 1, lanes 7–9) were analyzed by western blot for C3 levels. While there were slight differences in mobility of C3 α- and C3 β –chains between species, these data strongly suggest that human (Hu) and AGM serum C′ proteins are expressed at similar levels.

### Comparison of human versus AGM sera for C′-mediated neutralization of influenza virus

**Figure 2:**
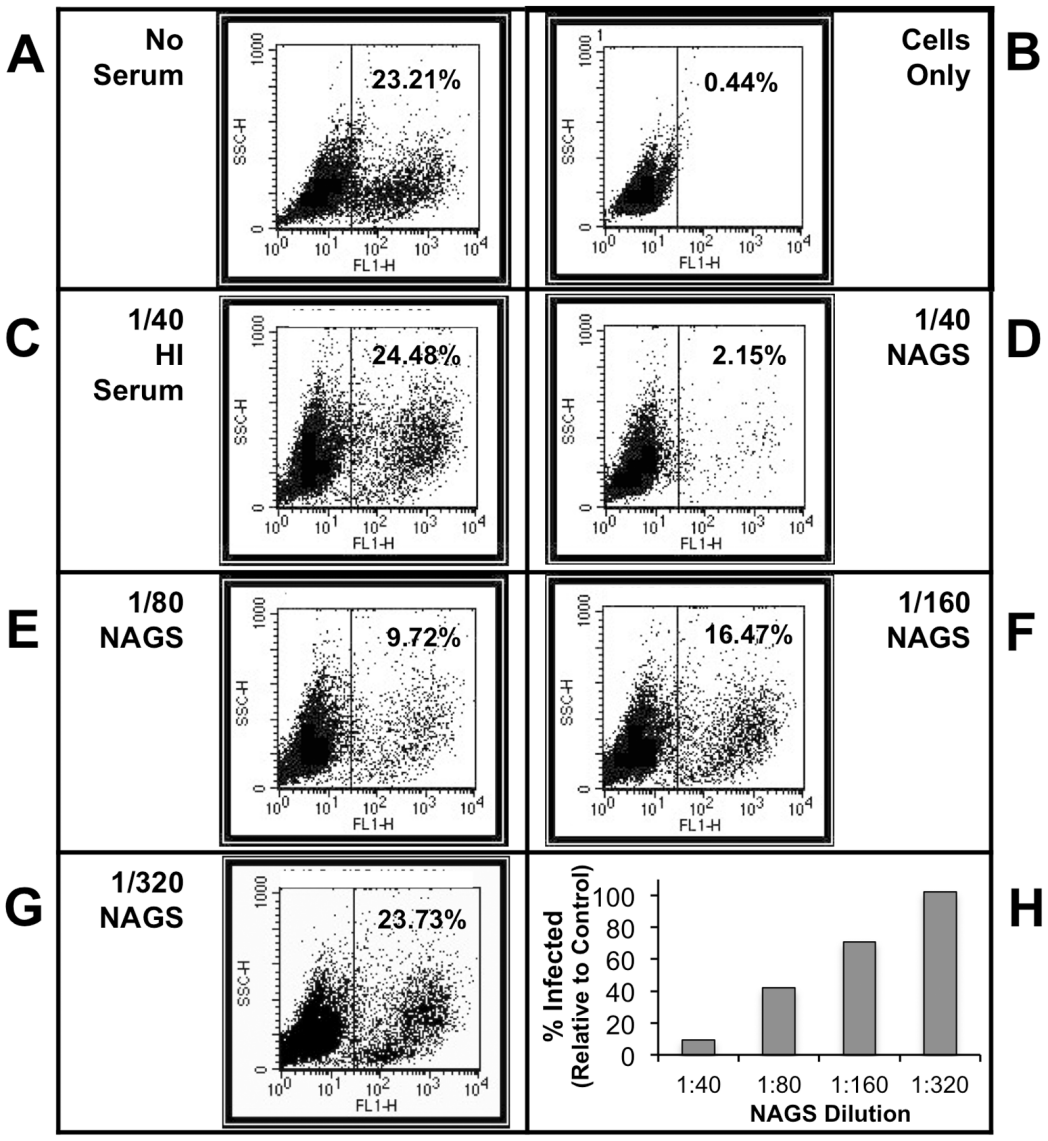
Flow cytometry-based PR8-GFP neutralization assay. PR8-GFP was mixed with a range of NAGS dilutions for 40 min at 37°C, and then used to infect U937 cells for 48 hr. Flow cytometry was used to determine the percentage of the cell population that was positive for GFP expression. Controls were: (A) Virus with no serum; (B) Cells only—no serum and no virus; (C) Virus plus 1∶40 HI AGM serum. AGM serum dilutions used to treat virus were: (D) 1∶40 NAGS; (E) 1∶80 NAGS; (F) 1∶160 NAGS; (G) 1∶320 NAGS. (H) Percent of cells that were GFP-positive following neutralization with indicated dilutions of NAGS from one example animal, expressed relative to that found for the no serum control.

A highly sensitive functional assay was developed for comparing the capacity of human and AGM serum to neutralize influenza virus *in vitro*. In this assay, dilutions of normal or heat inactivated (HI) human or AGM sera were mixed with a recombinant influenza virus PR8 that was engineered to express GFP as a fusion protein with the viral NS-1 protein [45]. After 40 min at 37°C, samples were used to infect U937 human monocytes. 48 h post-infection (pi) the percent GFP+ cells was determined by flow cytometry. As shown in Fig. 2, Panel A, virus in the absence of serum resulted in ∼23% of cells being infected. When virus was treated with a 1∶40 dilution of HI AGM serum (Fig. 2, Panel C), there was no reduction in infectivity. However, following treatment with a 1∶40 dilution of NAGS, only 2% of the cells were infected (Fig. 2, Panel D). This loss of infectivity with NAGS but not with HI AGM serum indicates that neutralization was dependent on intact C′ pathways. Further, PR8-GFP neutralization by NAGS was dose dependent, as treatment with 1∶80, 1∶160, and 1∶320 dilutions of serum resulted in 9.7, 16.5, and 23.7% of cells being infected, respectively (Fig. 2, Panels E, F, G). When expressed as a percent of infected cells seen in the no-serum-treated control, diluted NAGS showed a dose dependent neutralization of PR8-GFP with 1∶40, 1∶80, 1∶160, and 1∶320 serum dilutions corresponding with 9.3%, 41.9, 71.0, and 102.2% of cells infected, respectively (Fig. 2, Panel H).

**Figure 3:**
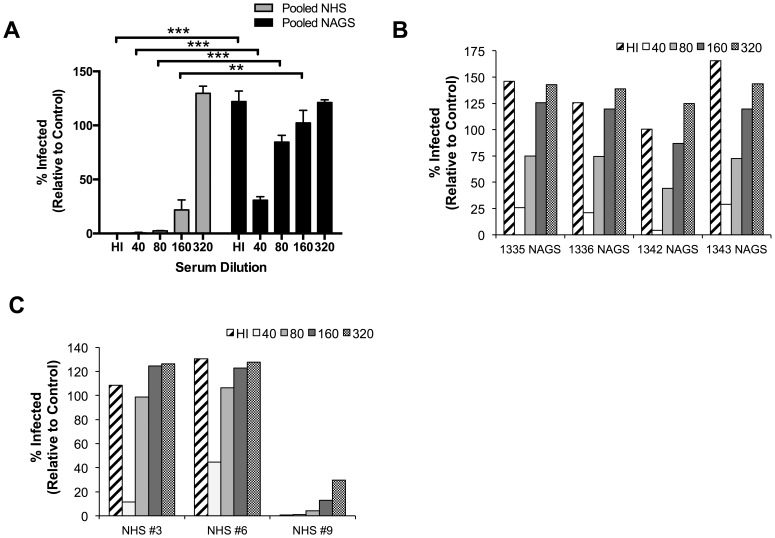
Comparison of C′-dependent neutralization of parainfluenza and influenza viruses by NHS and NAGS. (A) Influenza neutralization. PR8-GFP was incubated with the indicated fold dilutions of pooled Hu or AGM HI sera or varying dilutions of pooled NHS or NAGS for infection of U937 cells and analyzed as described in Fig. 2 (pooled samples are comprised of 4 individuals, Hu or AGM). Bars indicate mean of percent infected over triplicate experiments plus SD. ** p<0.001; *** p<0.0001 based on t-tests on log-transformed percentages for matching serum treatments for NHS and NAGS; treatments with no differences shown were not significantly different. (B) Variability in PR8 neutralization among samples from individual AGMs. PR8-GFP was incubated with the indicated fold dilutions of NAGS from four representative AGMs and analyzed as described in (A). There was no significant difference among animals based on a one-way ANOVA of log-transformed neutralization percentages. (C) Variability in PR8 neutralization among samples from individual Hus. Performed as in panel B, but with three individual Hus. Based on a one-way ANOVA of log transformed neutralization percentages, Hu subjects were significantly different from one another; p = 0.0045.

We hypothesized that neutralization of PR8-GFP with normal human serum (NHS) would occur in the absence of C′ due to pre-existing anti-influenza Abs, while NAGS neutralization would be C′-dependent because there are no circulating influenza viruses in the WFPC AGM colony. To test this hypothesis, we compared the capacity of NHS and NAGS to neutralize PR8-GFP using the flow cytometry assay described above. We found that neutralization of PR8-GFP by both pooled NHS and pooled NAGS is concentration dependent (Fig. 3A). However, NHS neutralized PR8-GFP more efficiently than NAGS—only 22% of the maximum infectivity was observed when treated with a 1∶160 dilution of NHS, while there was maximum infectivity following treatment with a 1∶160 dilution of NAGS (Fig. 3A). Importantly, we found that HI Hu serum neutralized PR8-GFP, but HI AGM serum did not (Fig. 3A). This indicates that influenza neutralization by pooled NHS can occur in a C′-independent fashion, while influenza neutralization with pooled NAGS requires C′.

To determine the variability in individual animals in the VRC, PR8 neutralization assays were performed using HI and NAGS samples. Results from four representative samples are shown in Fig. 3B. All animals showed dose-dependent anti-PR8 neutralization capacity. As expected, every HI serum sample tested lacked the capacity for PR8-GFP neutralization in the absence of C′ activity (Fig. 3B, cross-hatched bars), suggesting that these animals have no pre-existing anti-PR8 Abs. We also found that all of the animals tested had low variability of C′-mediated neutralization over the NAGS dilution series tested (Fig. 3B, data not shown). This demonstrates that NAGS does not have the pre-existing capacity to neutralize influenza virus in a C′-independent fashion.

In contrast, NHS samples are highly variable in this regard, likely due to variable previous exposure to any given strain of influenza virus. To demonstrate this, we performed the same influenza virus neutralization assay using NHS from three human subjects (Fig. 3C). Each of the three subjects demonstrated a different possible outcome. First, NHS #6 demonstrates the poorest neutralization of the three samples, where neutralization with a 1∶40 dilution of NHS or HI serum yields the highest percent of cells infected compared to the other to human samples. NHS #3 demonstrates improved neutralization, with a lower percent of cells infected after treatment with 1∶40 NHS than NHS #6 (Fig. 3C). Lastly, NHS #9 demonstrated a neutralization profile indicative of strain-specific Ab neutralization of influenza virus. While there was a dose-dependent effect of NHS #9 on viral neutralization, there was also C′-independent neutralization occurring, as indicated by 0.7% of cells infected by virus when treated with HI serum. This variability in Hu serum neutralization capacity makes it impossible to dissect the specific role of C′ during pregnancy using samples from Hu subjects.

### Comparison of PR8 neutralization by AGM pre- and post-immunization sera

To test the importance of neutralizing C′ in PR8-naïve versus PR8-immune animals, we inoculated two adult, non-pregnant AGMs with PR8 by a combined intra-nasal and intra-tracheal routes and collected serum samples from each animal at 14 d post-inoculation. In an identical experimental set up, we used real time PCR on tracheal washes collected two days after AGM inoculation with PR8 to confirm that PR8 replication can occur in the AGM respiratory tract (data not shown).

**Figure 4:**
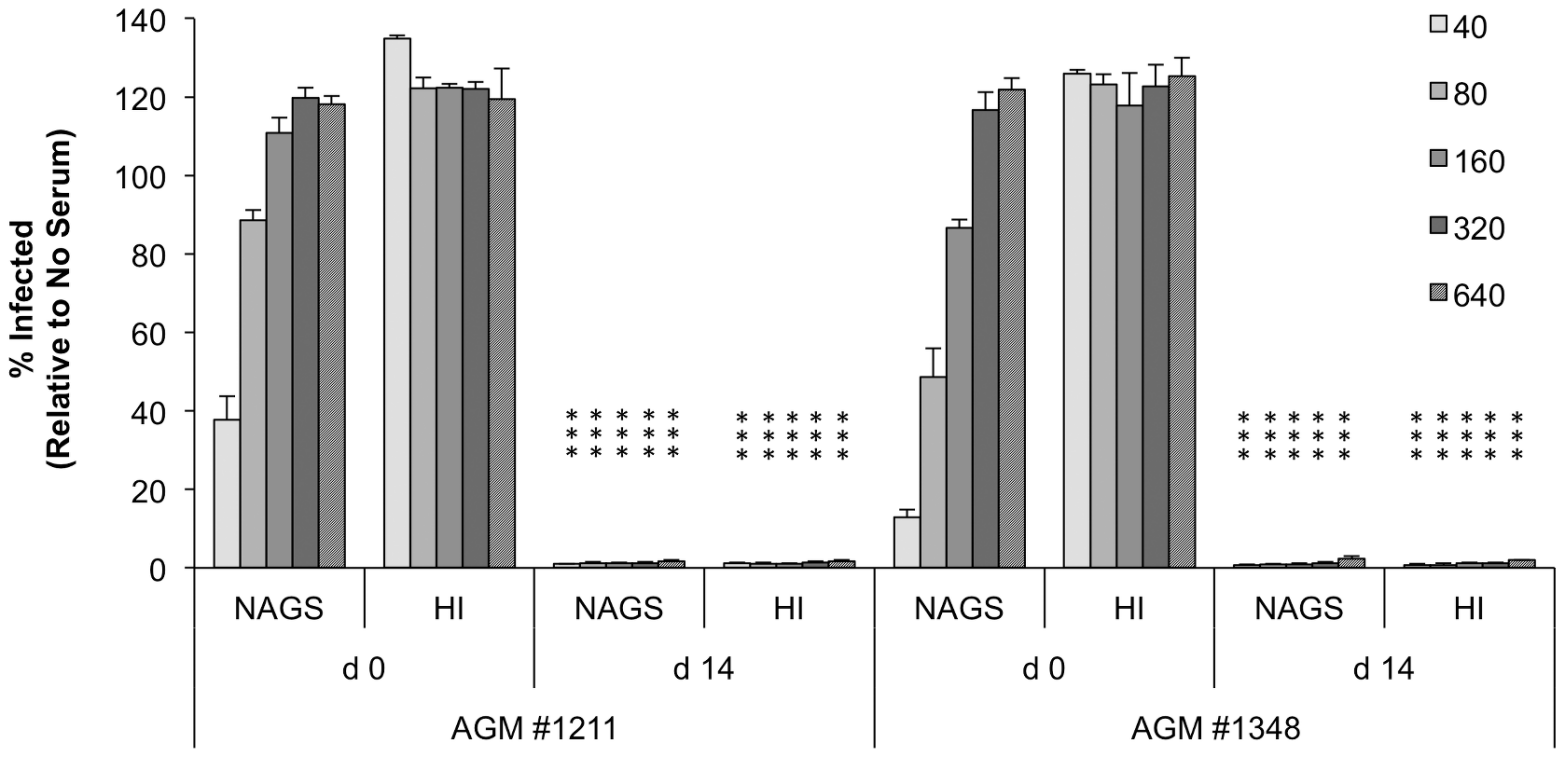
Effect of HI on the neutralization capacity of pre- and post-immune sera from AGM experimentally infected with PR8. Two AGMs were inoculated with PR8 as described in the Materials and Methods. Pre-immune sera (d 0) and sera collected at d 14 post-infection were tested at the indicated fold dilutions for *in vitro* neutralization of PR8-GFP as described in Fig. 2. Results are expressed as the mean plus SD from triplicate experiments to determine the percentage of GFP-positive cells compared to *in vitro* control infections (which lacked serum treatment). *** p<0.0001 as compared to matching serum treatment for the same animal at day 0 (shown on day 14 values) based on t-tests on log transformed neutralization percentages.

The capacity of sera to neutralize PR8 was tested using the PR8-GFP flow cytometry assay described above. As shown in Fig. 4, pre-immune sera showed dose-dependent neutralization of PR8-GFP and no neutralization was seen with HI sera. Interestingly, post-immune sera (normal and HI) were better at neutralizing PR8 than any Hu serum sample tested, as evidenced by complete neutralization of PR8-GFP out to a 1∶640 dilution (Fig. 4). The finding that PR8 was neutralized by HI post- but not pre-immune sera demonstrated that C′ is less important for *ex vivo* influenza neutralization after AGMs have been exposed to influenza virus.

**Figure 5:**
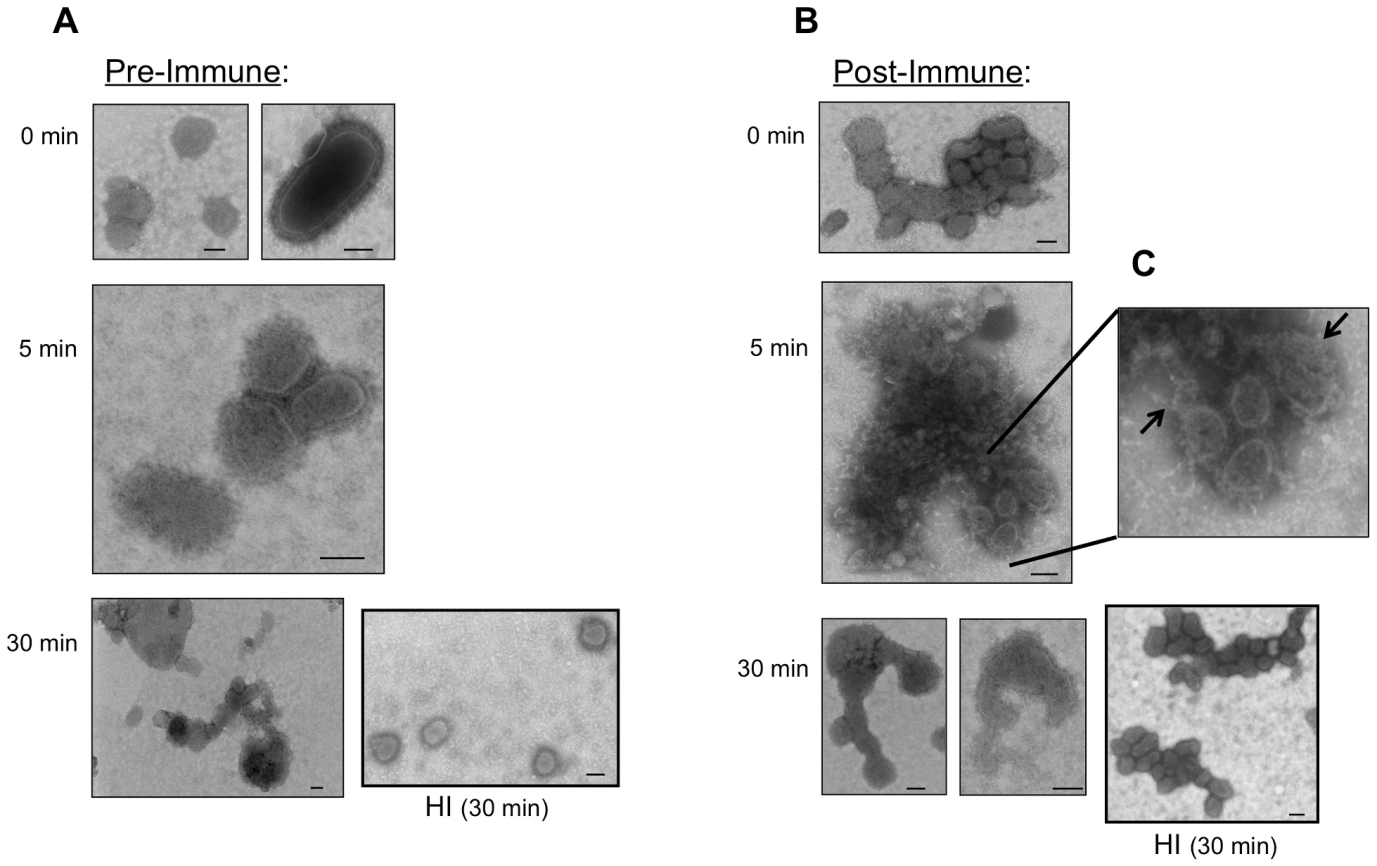
EM visualization of PR8 neutralization mechanism and kinetics by pre-immune, post-immune, and HI AGM sera. Sucrose-purified PR8 was incubated with a 1∶20 dilution of normal or HI (A) pre-immune or (B) post-immune AGM serum at 37°C for 0, 5, or 30 min, as indicated. These mixtures were fixed and negatively stained on a carbon-coated mesh grid and analyzed by EM. (C) Zoom of indicated area of post-immune 5 min time point with arrows indicating nucleocapsid leakage from lysed virus particles. Bar indicates 100 nm in each image, and magnification is as follows: (A) 0 min- 68,000x; 5 min- 68,000x; 30 min- 49,000× (B) 0 min- 49,000x; 5 min- 49,000x; 30 min- 49,000× (left); 98,000× (right).

C′-mediated virus neutralization can proceed by aggregation and/or lysis (Johnson, 2008). To determine the mechanism of virus neutralization by pre-immune AGM sera, we mixed PR8 and NAGS for various lengths of time and examined the virus structures by electron microscopy (EM) (Fig. 5). The PR8-serum mixture was kept on ice at time 0 to limit enzyme-driven C′ activity. Incubation with pre-immune serum resulted in intact, single virus particles at time 0 (Fig. 5A). After 5 min at 37°C, these pre-immune mixtures induced the formation of small, scattered aggregates of virus particles (Fig. 5A). By 30 min there were very few single virus particles apparent, but rather mid-sized aggregates, containing some lysed virus particles (Fig. 5A). Notably, after 30 min, treatment with HI pre-immune serum appeared to have no effect on virus morphology, as EM demonstrated scattered single, whole virus particles, similar to those visualized on the time 0 EM (Fig. 5A). These data indicate that C′-mediated neutralization of influenza virus with pre-immune sera proceeds through aggregation with only limited lysis at the dilution used.

In contrast, incubation with post-immune serum induced small virus particle aggregates as early as time 0 (Fig. 5B) and larger aggregates of lysed virus particles by 5 min, as evidenced by extensive nucleocapsid leakage (Fig. 5C, arrows). By 30 min there were no intact individual virus particles on the EM grid, rather they appeared only as scattered lysed virus particle debris (Fig. 5B). HI post-immune serum resulted in small virus particle aggregates but no lysis, similar to that seen on the time 0 grid (Fig. 5B). In summary, as compared with pre-immune serum, post-immune serum neutralization of PR8 involved rapid formation of large aggregates and substantial viral lysis. In the absence of active C′ (HI), post-immune serum resulted in virus particle aggregation without lysis. Thus, post-immune serum achieves neutralizing effects on virus in the absence of C′, while this was not the case with pre-immune serum.

### Decreased C′ neutralization capacity late in pregnancy

We sought to apply the above C′-mediated neutralization assays to the question of how C′ neutralization capacity changes during pregnancy. Serum samples were collected from 53 different animals at various time points during pregnancy and postpartum. As shown in Table 2, regardless of when during gestation an animal was identified as pregnant, it was most likely that we would capture a T3 sample using our sampling scheme. This late gestation bias in our sampling worked well for this particular study, as pregnant women are most susceptible to influenza late in pregnancy. For this reason, we chose to focus on samples from this group for our experiments.

**Figure 6:**
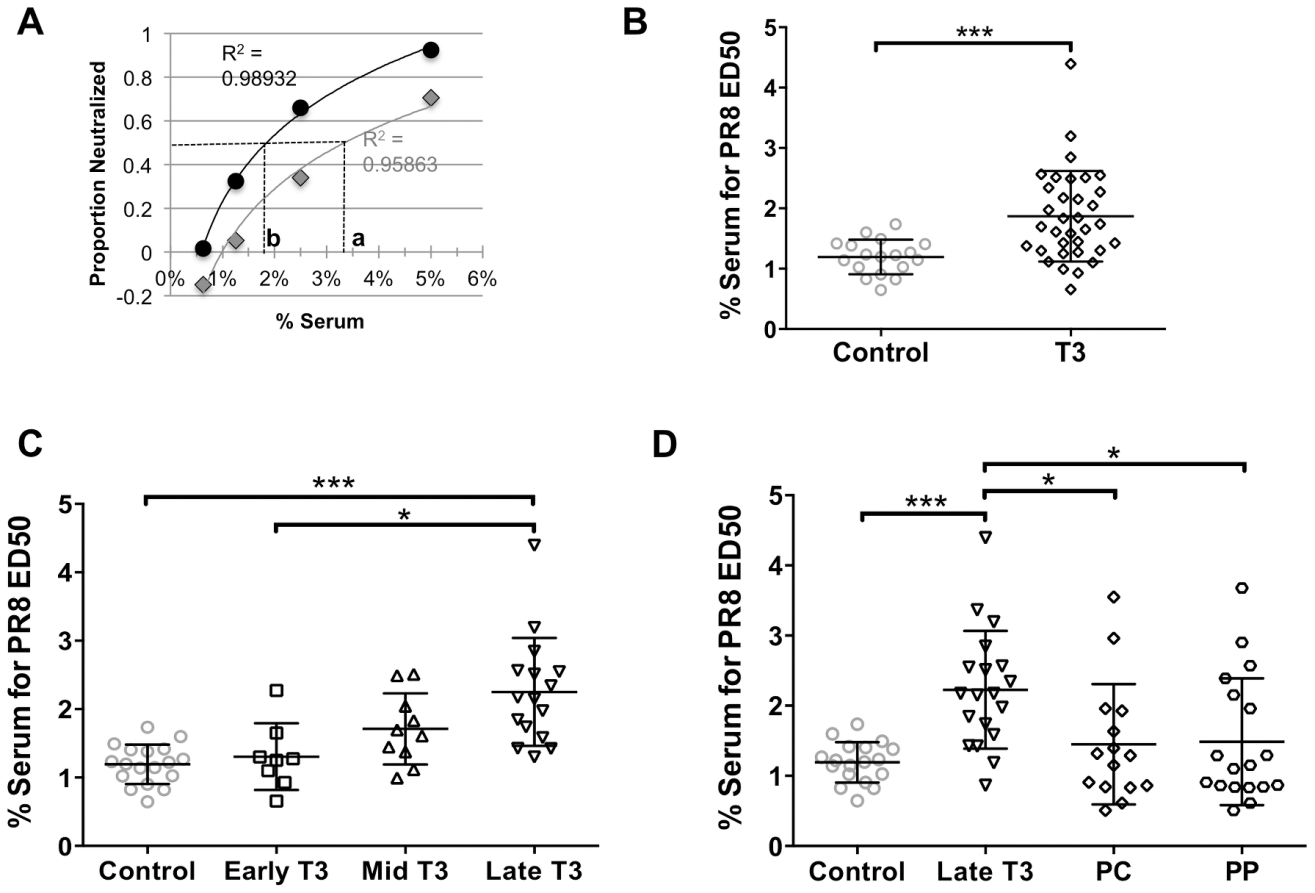
Capacity of T3 and postpartum serum C′ to neutralize PR8-GFP. C′ neutralization capacity was quantified as a function of the serum concentration effective in achieving 50% neutralization (ED_50_) for each animal. (A) Logistic regressions for two example animals, where dashed line crossing the x-axis (a or b) represents the ED50 for each animal; R^2^ is the coefficient of determination as an indication of how well the data points fit the regression model. ED50s were determined for AGMs at a range of gestation and postpartum dates: (B) T3 = 110–165 days; Shapiro-Wilks normality test indicated T3 population was not normally distributed; difference between T3 and control group tested with Mann-Whitney compare ranks two-tailed non-parametric test. (C) Early T3 = 110–128 days, Mid T3 = 129–146 days, Late T3 = 147–165 days gestation; (D) PC =  parturition check-in, 5–10 days after parturition; PP =  postpartum, 2–6 months after parturition. Differences among groups in panels C and D were tested with one-way ANOVA with Tukey correction for multiple comparisons. Lines within data point distribution represent mean +/−SD in panels B–D; * p<0.05; ** p<0.001; *** p<0.0001.

We tested the neutralization capacity of AGM sera collected at various times during pregnancy using the method described in Fig. 2. We quantified the percent of virus neutralized for a range of dilutions of sera and performed a logistic regression over the dilution series. For each animal at each pregnancy sampling date, we calculated the serum dilution that would be required to achieve 50% neutralization, or the *effective dose 50* (ED_50_). As shown for the two examples in Fig. 6A, a higher ED_50_ (i.e. where dashed line “a” crosses the x-axis for the grey series) indicates a poorer capacity to neutralize, and a lower ED_50_ (i.e. where dashed line “b” crosses the x-axis for the black series) indicates a better capacity to neutralize. The ED_50_ was determined for 34 serum samples collected from pregnant animals during their T3 and for 20 control (nonpregnant) females. For each serum sample, HI samples did not neutralize PR8-GFP (data not shown). As demonstrated by Fig. 6B, serum from T3 animals had significantly decreased capacity to neutralize PR8-GFP, as evidenced by the finding that ∼1.5 times the T3 serum was required to neutralize PR8-GFP compared to control animals.

We took advantage of our large sample size and full range of gestation dates and binned the T3 samples into smaller trimester tertiles (Fig. 6C). By comparing ED_50_ over early, mid, and late T3, we found that neutralization capacity is diminished in late T3 (p<0.05), but there is no significant difference between control sera and early or mid T3 sera (Fig. 6C). To determine how long after parturition this reduced neutralization capacity persisted, neutralization capacity at parturition check-in (PC, 5–10 days after parturition) and postpartum (PP, 2–6 months after parturition) was tested for the animals for which we had late T3 samples. Remarkably, we found that anti-influenza C′ neutralization capacity returned to control levels as early as 5–10 days postpartum (PC samples, Fig. 6D), and remained at control levels 2–6 months postpartum—that is, throughout and beyond lactation—(PP samples, Fig. 6D), which was the end of our study period. Further, the trend of decreased ED_50_ from late T3 to PC persisted when it was analyzed on an individual basis, utilizing our longitudinal sampling scheme (Fig. S1A). Likewise, there was no significant change in individual ED_50_ from PC to PP (Fig. S1B).

### Pregnant AGMs have decreased levels of serum C′ factors in late T3

**Figure 7:**
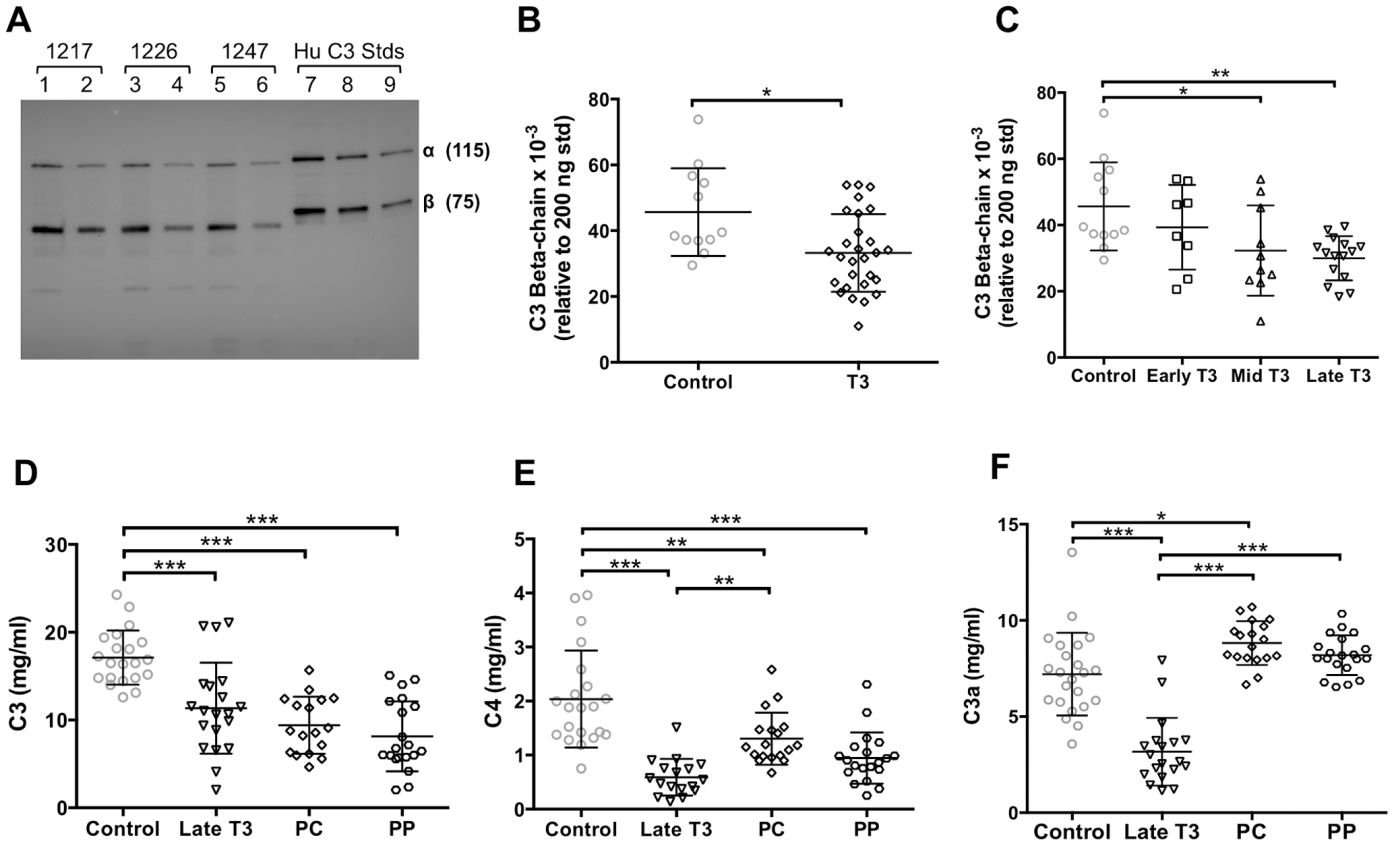
C′ factor quantification in T3 and postpartum AGMs. AGM serum C3 β-chain was quantified by western blot relative to purified Hu C3 standards. (A) Representative western blot with 1∶200 (lanes 1, 3, and 5) and 1∶400 (lanes 2, 4, and 6) serum dilutions shown sequentially for each of three different animals (1217, 1246, and 1247). Standards of 200 ng, 100 ng, and 50 ng of human C3 are in lanes 7, 8, and 9, respectively. (B) Quantification of serum C3 β-chain in T3 vs control animals, based on C3 β-chain band associated with 200 ng of standard Hu C3; difference between groups tested with unpaired two-tailed t-test. (C) Quantification of serum C3 β-chain across T3 tertiles (times defined in legend for Fig. 6C). ELISAs were used to quantify (D) C3, (E) C4, and (F) C3a, relative to purified human standards for each protein, during late T3 and at postpartum timepoints, as defined in the legend for Fig. 6D. Differences among groups in panels C–F were tested with one-way ANOVA with Tukey correction for multiple comparisons. Lines within data point distribution represent mean +/− SD in panels B–F; * p<0.05; ** p<0.001; *** p<0.0001.

One possible mechanism to explain the above decreased neutralization capacity in T3 is that there is a corresponding decrease in levels of C′ factors. To test this, C′ factor concentrations were quantified in the same serum samples as those used to determine ED_50_. Western blot analysis was used to determine circulating concentrations of AGM C3 β-chain, because this is the non-cleaved portion of C3 that remains intact regardless of activation state. Levels of AGM C3 β-chain were compared to the C3-β band associated with known quantities of human C3 (Fig. 7A). In accordance with decreased neutralization capacity shown above, there was a decrease in C3-β concentration in serum from T3 animals (Fig. 7B). When C3 β-chain concentrations were examined across early, mid, and late T3, we found that C3 β-chain concentrations were significantly decreased in samples from both mid and late T3 (Fig. 7C).

As an alternative approach, we developed ELISAs to quantify levels of C′ factors in AGM sera relative to purified human C′ standards. As shown in Fig. 7D, serum C3 concentrations were significantly reduced in late T3, consistent with the western blotting results above, and C3 concentration remained low up to 6 mos postpartum. Similarly, C4 concentrations were decreased in late T3 and remained low in PC and PP samples (Fig. 7E). As with C3 and C4, serum levels of the anaphylatoxin C3a were significantly decreased in late T3 (Fig. 7F). In contrast, however, C3a levels were increased in PC and PP samples (Fig. 7F), correlating with decreased ED_50_ (i.e. increased neutralization capacity) at those time points (Fig. 6D).

Our AGM sampling system enabled us to analyze large numbers of samples from animals over the whole course of the third trimester of the 5.5-month long gestation period, and to track individual animals longitudinally. As an example of this, one pregnant animal was sampled as part of a control group of (non-pregnant) animals one year later. The T3 C′ profile for this animal was compared to her “control” C′ profile as an example of an individual who fits the trends we established for the pregnant population (Fig. S2). This animal had reduced serum neutralization capacity, C3, C3a, and C4 in late T3 relative to one year later when she was a normally cycling, non-pregnant and non-lactating female in the VRC (Fig. S2).

## Discussion

In this study we tested the hypothesis that anti-viral C′ function is altered during pregnancy. To address this, we developed the AGM as a model for studies of C′ neutralization of influenza virus. The AGMs did not have detectable influenza virus neutralizing Abs, and therefore were most likely influenza-naïve. However, serum from influenza virus-naive AGMs neutralized PR8 through C′-dependent pathways by way of virus aggregation with limited lysis. Importantly, we showed that late in the third trimester of pregnancy, AGMs have significantly decreased serum C3, C3a, C4 and reduced influenza virus neutralization capacity relative to control animals. Taken together, our results demonstrate a late-pregnancy decrease in circulating C′ factors, which coincides with decreased C′-dependent neutralization of influenza virus—effects that correlate with the timing when pregnant women suffer severe influenza virus infections. This study is the first to demonstrate the effects of pregnancy on the C′ response to a human pathogen.

The role of C′ in protection from influenza virus infections is often overlooked. However, Jayasekera, *et al*. (2007) demonstrated that nIgM and C′ in mouse serum work together to neutralize influenza virus by forming coated viral aggregates, though nIgM or C′ alone were each insufficient to neutralize influenza virus. Our results suggest that if AGMs have circulating anti-influenza virus IgM, it also requires C′ to effectively neutralize influenza virus. This is based on our finding that sera from naïve animals neutralized influenza virus, but not when it was HI. Also similar to what Jayasekera, *et al.* demonstrated with naïve mouse sera neutralization, our studies found that PR8 formed aggregates with limited lysis in the presence of naïve AGM serum, but neither aggregation nor lysis was detected with HI sera.

Many aspects of an influenza virus infection are dramatically altered by the absence of C′ [23]. C3^−/−^ mice have delayed viral clearance and increased lung viral titers relative to WT mice, as well as reduced T cell priming in lung-draining lymph nodes and subsequent decreases in effector T cell number and function in the lungs. This suggests that if humans also display the decreased C′ levels and neutralization capacity that we observed late in AGM pregnancy, these changes may play a significant role in the increased burden of influenza illness suffered by women in late pregnancy. Additionally, C′ alterations may be an initiating and/or additive factor in other known pregnancy immune alterations, such as decreased Ig titers and a Th2 shift [2], [3], [49]–[52]. By extension, the role of C′ in other pregnancy infections associated with severe T3 disease such as measles, varicella-zoster, and hepatitis E virus, should be further explored [13].

Regulation of C′, and especially down-regulation of C′-mediated effects at the placental-maternal interface, is thought to be essential for fetal survival [53]–[55]. Deficiency in the rodent specific membrane-bound C′ inhibitory protein, Crry, led to mortality in 76% of neonates by 9.5 days post-coitus (dpc) and universal neonatal mortality by 16.5 dpc [56]. When mice lacked both *Crry* and *C3* genes, however, gestational viability was completely rescued, demonstrating that absence of C′ regulation directly results in neonatal death in mice [56]. Despite the clear importance of C′ in pregnancy maintenance, changes in the C′ system during human pregnancy are not completely understood.

A small number of studies have addressed changes in C′ levels during human pregnancy. One study indicated that pregnant women have increased plasma levels of the anaphylatoxins C3a, C4a, and C5a [39]. However, inflammatory effects of the anaphylatoxins were not measured, uncleaved C′ factors were not quantitated, and the pregnant women sampled were not synchronized in gestation date. Thus, time-dependent effects on C′-function may not have been evident. One of the only studies of longitudinal effects of pregnancy on C′ demonstrated a drop in serum C3 early in pregnancy, with a return to control or slightly elevated levels by second (T2) and third trimester (T3); this same study showed no difference in pathogen-independent C′-mediated hemolysis during these phases of pregnancy [37]. Lastly, a small study [38] found no significant changes in plasma C3 and C4 in individuals over the course of T3, though plasma C3 levels trended downward over the course of T3 in the study samples as a whole. This study, however, included no comparisons to samples from non-pregnant controls [38]. Most importantly, none of these prior studies used functional readouts for C′ control of infection. Thus, though past human studies have generated somewhat inconsistent results, the accepted paradigm is that pregnancy is, paradoxically, a state of C′ activation [37]–[39], [57].

There are several possible reasons why our findings may seem divergent from this model. First, it is possible that C′ regulation during AGM pregnancy is distinct from that during human pregnancy. We speculate that this is improbable given the high level of genetic relatedness between humans and old world monkeys and that the C′ system is highly evolutionarily conserved. More likely, the differences in study design and readouts between human studies and our work may present different aspects of complex C′ regulation during pregnancy.

We found decreased neutralization capacity and serum C′ protein concentrations in late T3 of AGM pregnancy. Studies indicating increased levels of C′ proteins during human pregnancy have largely focused on mid-T1 to mid-T3 [37], [39], [57]. Even so, several studies have shown trends of decreasing C3 and/or C4 late in pregnancy, which support our finding, albeit with a small number of samples that precludes conclusive analysis [37], [38], [57].

Comparing our findings to those of previous studies may also be problematic due to different sample preparations used in each study [37]–[39], [57]. Specifically, there are inconsistencies in use of plasma versus serum, which differ in their handling of coagulation factors, as well conditions for sample preparation (i.e clotting time and preparation temperatures). There are known interactions between coagulation factors and C′ factors C3, C4, and C5, which may lead to spurious activation of the C′ system [58], [59]. In addition, it is thought that pregnant women are in a hypercoagulable state due to increased levels of procoagulants and decreased levels of coagulation inhibitors [60]. Thus, the effect of coagulation factors, some of which remain in plasma but not serum, on C′ may be different for pregnant and nonpregnant women. In particular, this has the potential to skew concentrations of C′ activation products, such as C3a, C4a, and C5a. Finally, no prior human studies have examined functional response of pregnancy serum C′ to a pathogen. In this way, our finding here with neutralization of influenza may be a more clinically relevant readout than those previously reported.

C′ activation in the absence of infections would seem to be in direct conflict with physiologic needs of both the mother and the fetus during pregnancy, since C′ activation can promote inflammation, cell lysis, and anti-angiogenesis [33], [35]. Thus, while C′ plays a key role in protecting both the mother and fetus from potential infection [32], excessive C′ activation due to infection can contribute to disease pathogenesis and be very dangerous to the fetus, particularly late in pregnancy [61], [62]. The evolutionary balance between protecting the mother and fetus from infection, and protecting the fetus from the effects of C′ activation, might be expected to be tipped towards the latter as pregnancy progresses. Late in pregnancy, the mother has invested substantial energy resources into the fetus, and the fetus has a more developed immune system that is better equipped to respond to infection independently. Therefore, it might be most beneficial for the maternal C′ system to be down-regulated late in gestation. Our findings support this hypothesis, since we observed decreased C′ factors, decreased C3a anaphylatoxin (potentially secondary to decreased C3), and, most notably, decreased effectiveness in influenza virus neutralization late in T3.

Decreased influenza virus neutralization capacity in late T3 is the most striking and impactful finding from our study. Such changes, even at the magnitude of the 1.5- to 2.5-fold decreases we observed, have the potential to significantly alter clinical outcomes in at least three ways. First, complement is exceedingly potent—small amounts of C′ proteins can have large neutralizing effects on virus particles. Second, C′ activation products result in a sequestering effect and therefore contribute directly to vascular leakage, which enables serum proteins (including C′ proteins) to access sites of injury, such as infected lung tissue, at disproportionately high concentrations [63], [64]. Finally, the C′ cascade both self-amplifies and upregulates downstream immune responses, thus small changes can have exponentially larger downstream effects. In combination with our finding that, when in linear range, 2-fold dilutions of serum and its C′ factors can result in 3-times the number of PR8-GFP infected cells (Fig. 3B), the characteristics of the C′ system described here indicate that small changes in C′ concentrations and neutralization capacity can have both large (and amplifying) immediate effects on the severity of the viral infection, as well as alter the potency of the adaptive immune response.

Though small changes in C′ are widely accepted to have large effects on response to infection [65], to our knowledge the magnitude of this effect has not been previously reported. For similar reasons, it is impossible to interpret the variation in neutralization capacity seen within individuals during the postpartum period we tested (Fig. S1). While there were not significant changes across the whole group, individual changes during this time may result in changes in severity of viral infection at the individual level. Future studies using our pregnant AGM model could provide the first natural system in which the effects of small changes in C′ during viral infection are quantified.

One possible mechanism for decreased virus neutralization capacity late in T3 is dilution of C′ factors (and therefore diminished neutralization capacity) due to the 40–50% increase in blood volume that occurs during pregnancy [66]. In humans, return to normal blood volume occurs within approximately two weeks after birth [67], while other blood elements (such as RBC volume) return to normal over the course of 8 weeks after birth [68]. Yet we observed no difference in C′ neutralization capacity between PC (5–10 days postpartum) and PP (2–6 months postpartum) samples, and C3 and C4 remained lower than in control animals at both of these time points. This indicates that the decreased C′ factors and neutralization capacity that we observed late in pregnancy are regulated by means beyond simple blood dilution. During pregnancy, capacity to neutralize influenza virus may be diminished because there are fewer circulating C′ factors, but functional activity of circulating C′ factors could also be downregulated. Likewise, neutralization capacity may improve postpartum, despite low C′ factors, if C′ potency improves. An alternative explanation might be that nIgM plays a larger role in neutralization postpartum than it does during pregnancy—also possibly due to increased concentration or potency.

Interestingly, in contrast to low postpartum C3 and C4 levels, we observed increased postpartum C3a levels. This finding might be explained by one or a combination of the following: 1) C′ is highly activated postpartum, causing cleavage of C3 and an abundance of the anaphylatoxin C3a in serum; 2) new C3 and C4 are not being produced adequately to replace C3/C4 depleted during C′ activation, possibly due to loss during lactation [69]; 3) C3a clearance is reduced due to decreased expression of C3a receptors or inhibition of other downstream events. Regardless of the mechanism, C′ factors, and therefore C′ activity and C′ neutralization capacity, appear to be tightly regulated in a temporal and complex fashion during pregnancy. This regulation undergoes drastic alterations at the time of parturition, as demonstrated by changes in neutralization capacity and C3a by 5–10 days after parturition (Fig. 6D and 7F).

One possible mechanism by which C′ alterations might occur during pregnancy and postpartum is through changes in sex hormones such as progesterone and estrogen. Both of these sex hormones peak during pregnancy at 20–30 times normal peak cycling levels; at parturition they return precipitously to normal levels. Prior work has shown that estrogen administered at pregnancy concentrations to non-pregnant mice is sufficient to recreate the abrogated innate immune response to influenza virus infection observed in pregnant mice [70]. However, C′ levels and C′ function were not examined in this study. Interestingly, there is a known estrogen response element (ERE) in the promoter of C3 [71], thus providing one possible mechanism by which sex hormones could alter C′ levels and its capacity to respond to viral infection. Given that female AGM cycling hormone levels match those of humans, the AGM model would be ideal to address these questions.

NHPs are increasingly appreciated as strong model systems for examining influenza virus pathogenesis and immune response [72], [73]. NHPs infected with influenza virus exhibit clinical signs similar to those of humans including fever, malaise, nasal discharge, and cough; additionally, virus replication can be detected in the nasal passages and respiratory tract [74], [75]. They are particularly valuable experimentally because captive NHPs in research colonies are generally influenza-naïve. While many previous NHP influenza virus studies have utilized macaques, there is no strongly established nonhuman primate model for reproductive physiology or immunity during pregnancy. That is a novel aspect of our work. Cycling, sexual and mating behavior, and the absence of a true “breeding season” make the AGM a more comprehensive model for human reproductive physiology than the macaque provides for this developing field. Finally, we have access to a robust AGM breeding colony, which can serve as a precious resource for addressing such questions.

Thus, pregnant AGMs model the scenario where pregnant women are infected with an emergent seasonal or pandemic influenza strain. This is a valuable system for studying the effects of changes in C′-mediated neutralization during an experimental primary infection or vaccination scenario, or with differing primary and secondary infections to study the role of C′ in cross protection. Though many reagents and laboratory methods have been developed specifically for macaques, AGM-specific reagents are less common. Thus, our work also exemplifies the process of optimization of existing reagents for a different NHP species. However, the AGM genome has been sequenced and is in the process of being annotated. Because of this, it is likely that in the near future, an increasing number of AGM-specific reagents may be developed.

Likewise, longitudinal approaches afforded by NHP colonies could be particularly useful for studies of infant mortality, stillbirth, aborted pregnancy, and other poor pregnancy outcomes. For example, the findings reported here could be used as a baseline for comparing C′ function in AGM pregnancies with poor outcomes (i.e. stillbirth, aborted fetus), rather than simple serum or plasma protein measurements that do not consistently correlate with pregnancy outcomes [36], [38]. AGM breeding colonies have poor pregnancy outcomes at rates approximating those among humans, and they provide access for tracking, assessment, and follow-up studies in these circumstances. Further, due to the multi-generational nature of breeding colonies, alterations in innate immune response among certain animals and lineages could be traceable to genetic differences. As genome-wide association studies (GWAS) become increasingly accessible and more commonly performed, we may be able to identify clinically relevant single nucleotide polymorphisms (SNPs) in genes for C3 or other C′ factors. In future studies, the AGM model system we have developed will enable us to both determine the role of physiologic and hormonal changes in downregulating C′-mediated immunity during pregnancy and permit the identification of therapeutic targets to improve viral infection outcome in pregnant women.

## Supporting Information

Figure S1 **Changes in capacity of serum C′ to neutralize PR8-GFP for individual animals over time.** Serum anti-PR8-GFP ED_50_ was quantified, as described in legend to Fig. 6, and tracked for 14 individual animals over time (A) from late T3 to PC and (B) from PC to PP. Solid lines represent negative slopes and dotted lines represent positive slopes in ED_50_ over time with animal numbers indicated in panels A and B. (C) Table of information about the 14 animals that were tracked over time in A and B; no correlation found between changes in ED_50_ and the pregnancy traits listed here. Two-tailed Wilcoxon matched-pairs signed rank test was performed for A and B, where * p<0.05, no difference shown indicates no significant difference over individual pairs.
(TIF)

Figure S2 **C′ neutralization capacity and serum C′ factors in one AGM during T3 and one year after pregnancy.** Neutralization capacity (A) and serum C3, C3a, and C4 concentrations (B) for AGM 1375 late in T3 (black bars) were compared to one year later when 1375 was a non-pregnant female “control” (grey bars). n = 1 animal; bars represent mean and SD of technical triplicates.
(TIFF)
