# Supplementary figures and images for: An AGM Model for Changes in Complement during Pregnancy: Neutralization of Influenza Virus by Serum Is Diminished in Late Third Trimester
Source: PLoS One. 2014 Nov 19;9(11):e112749. doi: 10.1371/journal.pone.0112749 (PMC4237339; doi:10.1371/journal.pone.0112749)

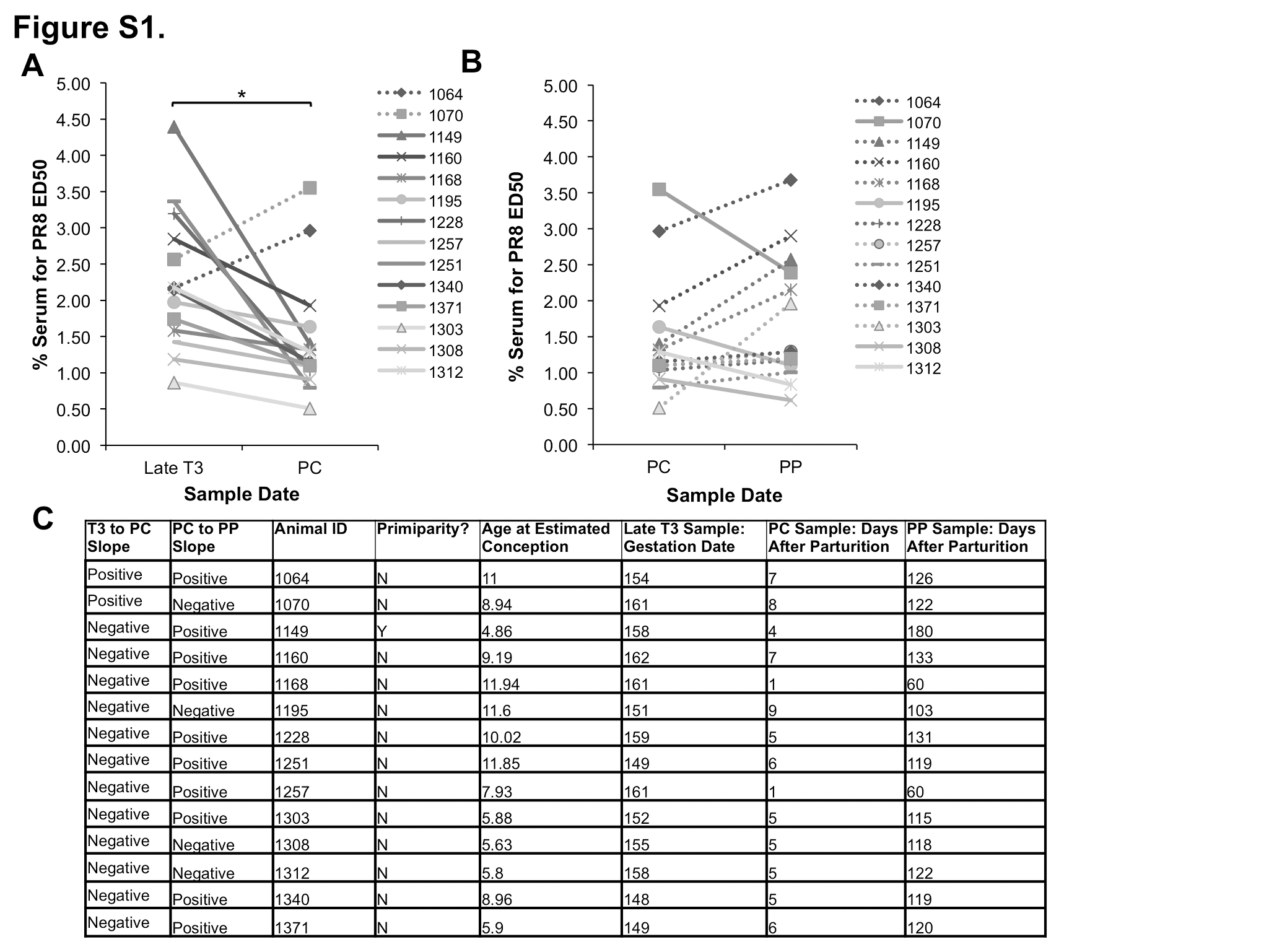

Supplement: Figure S1 — Changes in capacity of serum C′ to neutralize PR8-GFP for individual animals over time. Serum anti-PR8-GFP ED50 was quantified, as described in legend to Fig. 6, and tracked for 14 individual animals over time (A) from late T3 to PC and (B) from PC to PP. Solid lines represent negative slopes and dotted lines represent positive slopes in ED50 over time with animal numbers indicated in panels A and B. (C) Table of information about the 14 animals that were tracked over time in A and B; no correlation found between changes in ED50 and the pregnancy traits listed here. Two-tailed Wilcoxon matched-pairs signed rank test was performed for A and B, where * p<0.05, no difference shown indicates no significant difference over individual pairs. (TIF) [file pone.0112749.s001.tif]

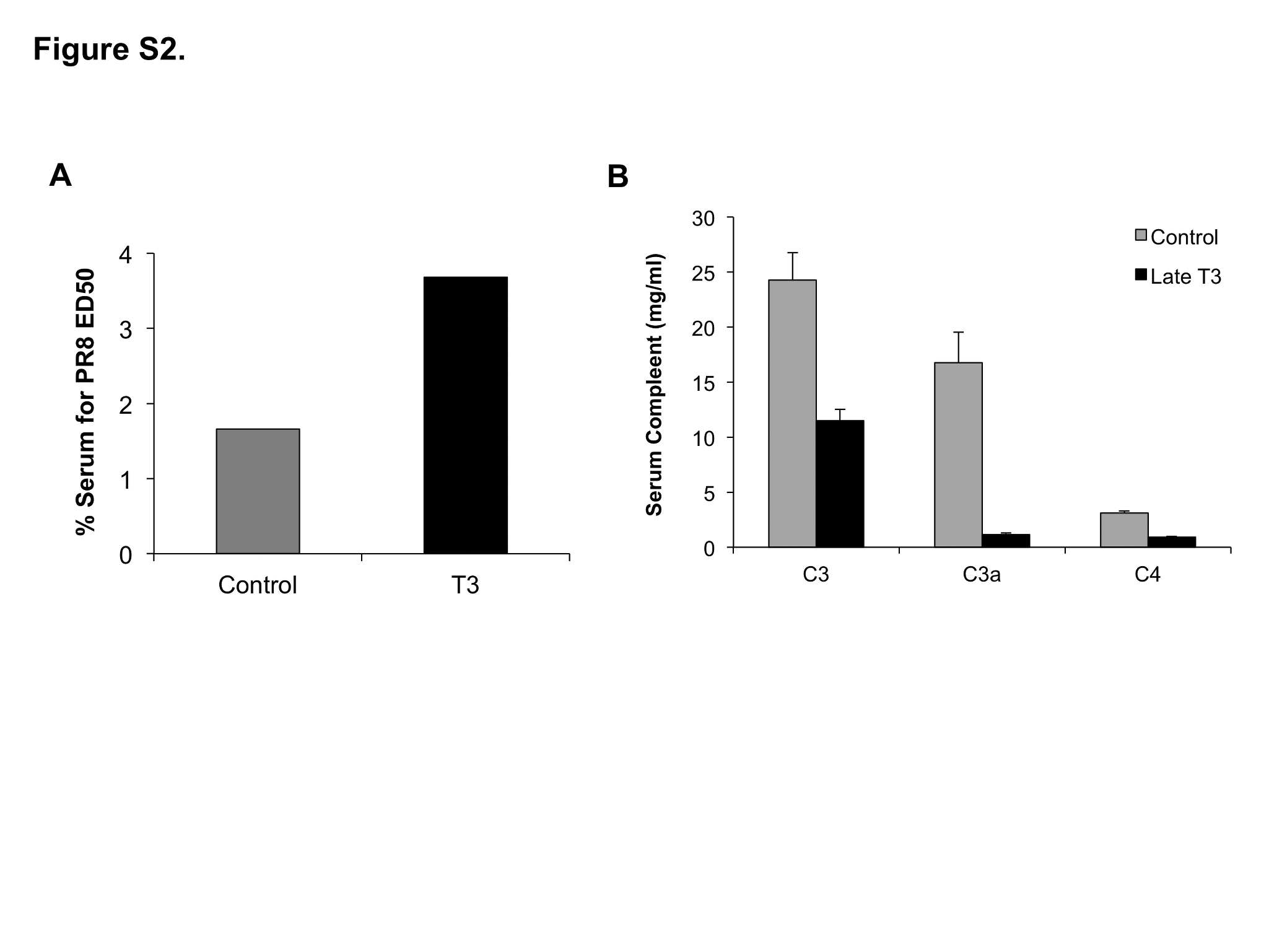

Supplement: Figure S2 — C′ neutralization capacity and serum C′ factors in one AGM during T3 and one year after pregnancy. Neutralization capacity (A) and serum C3, C3a, and C4 concentrations (B) for AGM 1375 late in T3 (black bars) were compared to one year later when 1375 was a non-pregnant female “control” (grey bars). n = 1 animal; bars represent mean and SD of technical triplicates. (TIFF) [file pone.0112749.s002.tiff]
